# Foot Morphological Difference between Habitually Shod and Unshod Runners

**DOI:** 10.1371/journal.pone.0131385

**Published:** 2015-07-06

**Authors:** Yang Shu, Qichang Mei, Justin Fernandez, Zhiyong Li, Neng Feng, Yaodong Gu

**Affiliations:** 1 Faculty of Sports Science, Ningbo University, Ningbo, China; 2 Department of Engineering Science, University of Auckland, Auckland, New Zealand; 3 Auckland Bioengineering Institute, University of Auckland, Auckland, New Zealand; 4 School of Biological Science and Medical Engineering, Southeast University, Nanjing, China; 5 Rehabilitation Center, Ningbo Ninth Hospital, Ningbo, China; Bern University of Applied Sciences, SWITZERLAND

## Abstract

Foot morphology and function has received increasing attention from both biomechanics researchers and footwear manufacturers. In this study, 168 habitually unshod runners (90 males whose age, weight & height were 23±2.4years, 66±7.1kg & 1.68±0.13m and 78 females whose age, weight & height were 22±1.8years, 55±4.7kg & 1.6±0.11m) (Indians) and 196 shod runners (130 males whose age, weight & height were 24±2.6years, 66±8.2kg & 1.72±0.18m and 66 females whose age, weight & height were 23±1.5years, 54±5.6kg & 1.62±0.15m)(Chinese) participated in a foot scanning test using the easy-foot-scan (a three-dimensional foot scanning system) to obtain 3D foot surface data and 2D footprint imaging. Foot length, foot width, hallux angle and minimal distance from hallux to second toe were calculated to analyze foot morphological differences. This study found that significant differences exist between groups (shod Chinese and unshod Indians) for foot length (female p = 0.001), width (female p = 0.001), hallux angle (male and female p = 0.001) and the minimal distance (male and female p = 0.001) from hallux to second toe. This study suggests that significant differences in morphology between different ethnicities could be considered for future investigation of locomotion biomechanics characteristics between ethnicities and inform last shape and design so as to reduce injury risks and poor performance from mal-fit shoes.

## Introduction

Barefoot running has received increasing attention in recent years. From the perspective of evolutionary theories, long-distance running ability was crucial for human survival [1,2]. Several previous studies were conducted to investigate the difference between habitually barefoot runners and shod runners (with shoes) concerning different foot-strike patterns with foot strike angle or strike index analysis [2–4], kinetics of running, walking and jumping for injury risks evaluation [5–7], and muscle activity characters of the lower limb [8]. However, biomechanical analysis of barefoot or shod running has not led to agreement on which running style is more injury-preventive or running-economic. Barefoot running was popularized with enhancement of proprioceptive motor-regulation function and muscle strength, especially medial gastrocnemius (MG) of barefoot runners for ankle plantar-flexion [9,10] and thus help prevent repetitive stress injuries, like tibial stress fracture and patellofemoral pain syndrome [11]. In contrast, running with shoes was believed to reduce the loading rate and plantar pressure to the lower extremity and foot, especially the athletic footwear equipped with cushioning system [12], with propagated ‘minimalist’ shoes showing non-convincing effect of perceptible barefoot feeling, lowering injury risk or increasing running economy [6,13–16]. Reasons might be that there exists different running styles or techniques (with the forefoot striking pattern) rather than simple barefoot running (without shoes) [17,18]. Also, footwear has been shown to influence foot morphology as measurement of different foot structure [19–22], particularly incorrectly fitted shoes, the feet binding of Chinese women [23] and hallux valgus of women owing to long-term wearing of high-heeled shoes [24]. Even wearing normal shoes from a young age may influence the shape of feet compared with habitually barefoot populations. It has been shown that there are a multitude of differences in foot type, foot pressure or loading and foot morphological characteristics among people of different genders, ages and ethnicities [25–30].

Different morphological foot characteristics are associated with different functions. The normal foot with 26 bones and associated muscles ensures the foot’s static and dynamic functions and contributes to the overall features of the foot [26], but the shape and morphology differs from individuals [31,32]. Knowing exactly the functions of different feet morphology not only plays a crucial role in preventing injuries [33,34], but also informs sport performance [35,36]. Highly competitive and recreational athletes are at risk of incurring a wide range of injuries, typically hyperkeratotic lesions like corns and calluses [37], or stress induced injuries [38,39]. One widely accepted explanation was that the lack of protection provided by sports shoes [33] or ill-fitted shoes [40,41] leads to injuries and reduced performance [42]. Different foot morphology has become a focus in order to reduce injury when designing shoes [43]. When it comes to anthropometry of human feet, indexes like length, width and girth or circumference of specific feet regions have been collected and utilized in footwear design since the introduction of traditional anthropometric methods [44]. Studies have been conducted to confirm the reliability and reproducibility of foot type or morphology measurement systems compared with traditional methods both under static and dynamic conditions [21,44–47].

The reported morphological difference between habitually unshod and shod populations were that unshod feet are wider than shod feet [48] and shod walkers have slender feet (short and narrow) compared with unshod walkers. Compared with habitually shod feet, the big toes of habitually unshod feet are quite separate from the other four toes, which was believed to be the toes’ prehensile function like fingers [30]. Quantified indices have not been used to illustrate the toes morphological difference between habitually shod and habitually unshod feet. The hallux angle (HA) is the angle created by the deviation of the hallux (Line _B-C & B’-C’_) away from the tangential line, which connects the medial heel with the medial forefoot (Line _A-B & A’-B’_) [35]. In this study, the primary objective was to quantify the hallux angle, minimal distance between hallux and the interphalangeal of the second toe (D), foot length and foot width between habitually unshod and habitually shod runners of different ethnicities. The minimal distance between the hallux (big toe) and the interphalangeal joint of the second toe is depicted based on what was collected for this study. Feet deformities, like hallux valgus, were excluded for its influence on the hallux angle owing to the long-term wearing of ill-fitted shoes [48]. The hallux angle and minimal distance between hallux and toes may provide additional indices to quantify differences between shod and unshod feet. The secondary objective was to evaluate any association between the hallux angle and minimal distance of habitually unshod and shod feet to identify any morphological trends.

## Materials and Methods

### Ethics statement

This study was approved by the Ethics Committee of Ningbo University. Before the test experiments, the subjects were informed of requirements and procedures of the scanning test. All gave informed written consent to participate in the study.

### Participants

A total of 364 participants, including 168 habitually unshod runners (Indians) and 196 shod runners (Chinese) volunteered to take the foot scan test. The Indian unshod runners were chosen from over one thousand International students in Ningbo University while conducting physical examinations; and the Chinese shod runners were native undergraduate students of Ningbo University. All participants had a history of running outdoors or on treadmills and kept participating in physical activities at least three times a week for an hour each time. The Indian unshod runners originated from South India, who were barefoot running or taking part to physical activities since born and wore slippers or flip-flops in daily life. The Chinese shod runners wore shoes since born and kept wearing different kinds of shoes in daily life. Participants who presented hallux valgus, high-arched foot, flat foot, diabetic foot or any other foot deformities were excluded by physical examiners while participating physical examinations before the scanning test. All participants had no injuries or surgeries to their lower limbs in the past half year. Their basic demographics are listed in Table 1.

**Table 1:** The basic demographics of habitually unshod and shod runners.

	Unshod runner		Shod runner	
	Male	Female	Male	Female
**Number**	90	78	130	66
**Age(yrs)**	23±2.4	22±1.8	24±2.6	23±1.5
**Weight(kg)**	66±7.1	55±4.7	66±8.2	54±5.6
**Height(m)**	1.68±0.13	1.6±0.11	1.72±0.18	1.62±0.15
**BMI(kg/m** ^**2**^ **)**	23.38±1.11	21.48±1.12	22.31±1.75	20.57±1.69

Note: Mean±Standard Deviation; BMI-body mass index.

### Methods and equipment

**Fig 1:**
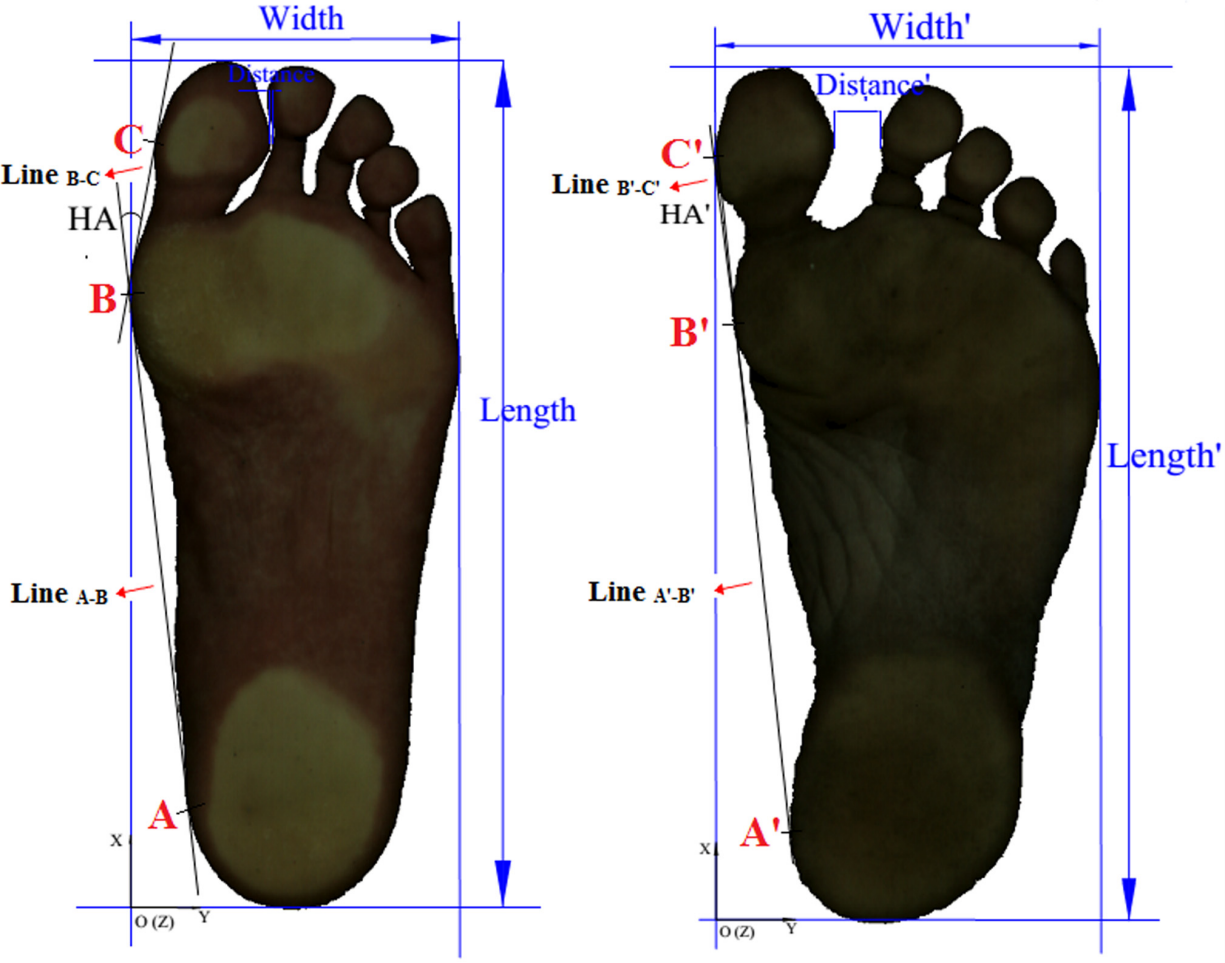
2D foot print image of habitually shod (left) and unshod (right) runners.

**Fig 2:**
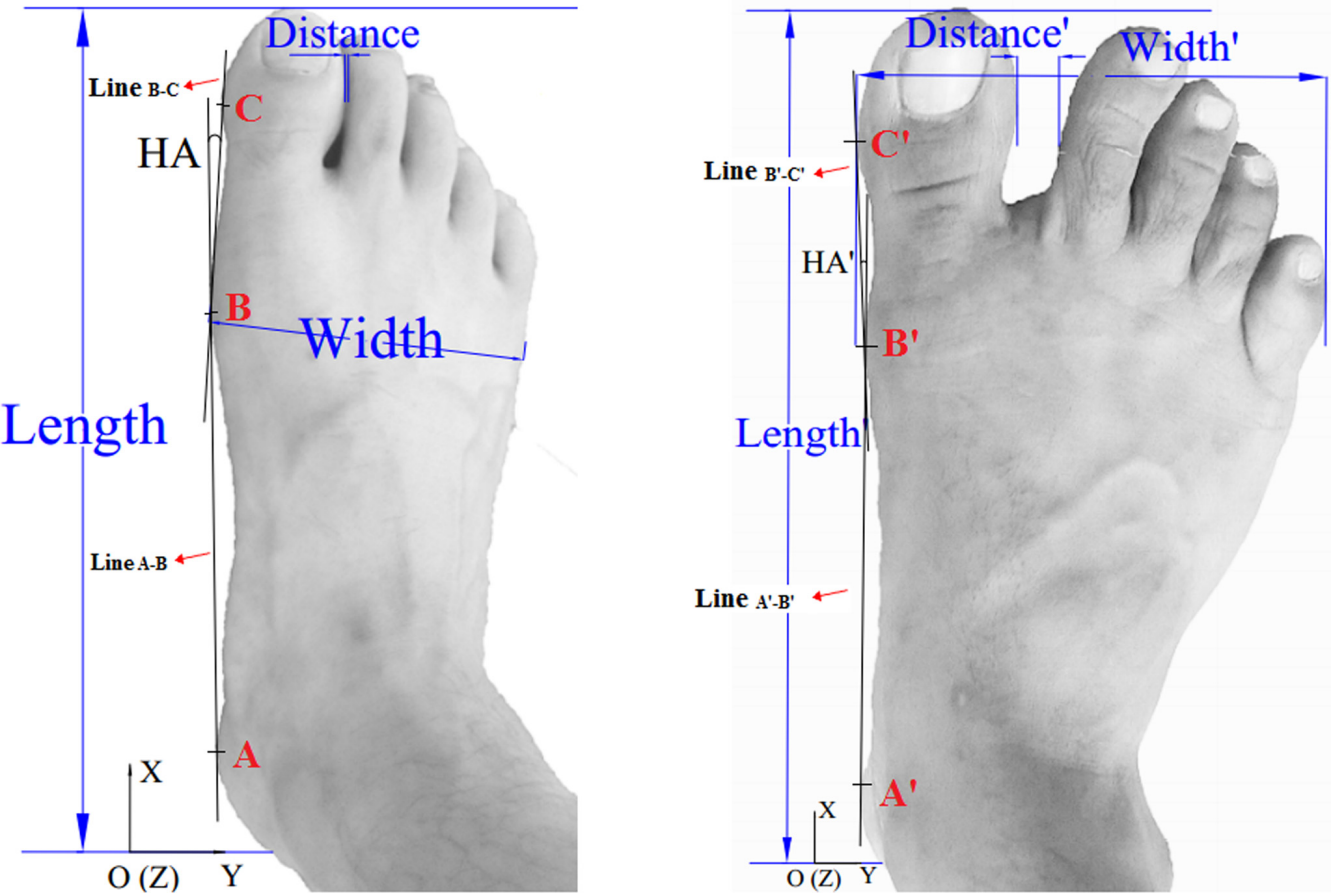
The dorsal view of foot surface data, length (length’), width (width’), minimal distance (distance’) and HA (hallux angle, HA’). Three landmarks were drawn to connect line A-B (A’-B’) and line B-C (B’-C’), with A (A’) in medial calcaneous, B (B’) in the head of the first metatarsophalangeal joint and C (C’) in the hallux.

The Easy-Foot-Scan (EFS), OrthoBaltic (Kaunas, Lithuania) was utilized to process and acquire the 3D foot surface data and 2D foot print image simultaneously. The scan speed, scan sensitivity, resolution, smoothing and hole filling of EFS in the measuring interface were set at fast, normal, 1.0mm, 30mm and 100mm, respectively. To accurately obtain the 3D data and 2D image, the procedure strictly followed the international standard, ISO (International Standards Organization)-20685 and 7250 [49]. As noted by Telfer and Woodburn [49], these standards have ‘been produced with the aim of ensuring that measurements taken using 3D scanning systems are comparable with those taken using traditional methods and can be used in anthropometric databases.’ These standards ‘require that the maximum mean difference between the traditional and 3D scanning derived values is 2 mm.’ The EFS system in this scanning test is equipped with a high precision of 0.3 mm. For the calculation of hallux angle, three landmarks were previously hand-drawn to the medial calcaneous (A & A’), the head of the first metatarsophalangeal joint (B & B’) and the hallux (C & C’) for each participant (S2 Fig). Two lines (line _A-B & A’-B’_ & line _B-C & B’-C’_) were used to calculate the hallux angle (HA and HA’) in Auto CAD (Computer Aided Design, 2007) and the minimal distance (D and D’) between the hallux and interphalangeal joint of the second toe computed from the 2D foot print image (Figs 1 and 2).

Participants were asked to stand still with their right foot in the middle of the glass plate (scanning area) and left foot on the supporting plate outside the scanning area (S1 Fig). The distance between the two feet is the width of their shoulders so that the participants’ body weight can be evenly distributed to both feet (S1 Fig). The BMI (body mass index) is defined as the body weight (kg) divided by squared body height (m^2^). The World Health Organization (WHO) defines BMI values between 18.5 and 24.9 as normal; values below 18.5 as underweight and values over 30 as obese [50]. The BMI of participants was in the normal range between 18.50 and 24.99 kg/m^2^ [51], seen in Table 1. As the BMI of all participants were in the normal range, the foot shape changes for different body weight or load-bearing conditions and different stature can be disregarded under the condition of bearing their own body weight [51–54].

### Data acquisition and statistical analysis

To abide by the ISO 20685 and 7250 standards, the 3D surface data collected in the test was limited to measuring results of foot length and width, excluding ball perimeter, waist girth perimeter, instep heel perimeter, short heel perimeter, ankle circumference perimeter and skin circumference perimeter. The hallux angle (Figs 1 and 2) is the angle created by the deviation of the hallux away from the tangential line which connects the medial heel with the medial forefoot [35]. The 2D foot print images (Figs 1 and 2) were collected to calculate hallux angle (HA&HA’) value and the minimal distance (D&D’) between hallux and the second toe with Auto CAD 2007 (Autodesk, America).

All statistical analysis was performed using the software SPSS version 17.0 (SPSS Inc., Chicago, IL, USA). The one-way ANOVA (analysis of variance) was taken to analyze the significance of length and width difference between habitually unshod (Indian) and shod (Chinese) feet. The LSD (least significance difference) in ANOVA was conducted to analyze the significance of hallux angle and difference between habitually unshod and shod feet. The significant p-value was set at 0.05.

## Result

The length, width, hallux angle and minimal distance between hallux and the interphalangeal joint of the second toes of habitually unshod (Indian) feet and habitually shod (Chinese) feet were collected and analyzed to quantitatively show foot morphological characteristics. The individual level foot morphology data collected is shown in the S1 Table.

### Length and width of unshod feet and shod feet

As shown in Tables 2 and 3, the length and width of habitually unshod feet and habitually shod feet are divided into different feet length and width sample distributions. The age, weight and height or BMI of all participants in the test are presented in Table 1. They are classed in similar age and BMI group.

**Table 2:** The length sample distribution of unshod feet and shod feet.

Unit: (mm)		<220	220–230	230–240	240–250	250–260	260–270	270<	Sum.
Unshod feet	Male	0	3(3.3%)	3(3.3%)	12(13.4%)	54(60%)	6(6.6%)	12(13.4%)	90
	Female	0	6(7.7%)	36(46.2%)	24(30.8%)	12(15.3%)	0	0	78
Shod feet	Male	0	0	8(6.2%)	28(21.5%)	40(30.8%)	28(21.5%)	26(20%)	130
	Female	4(6.1%)	10(15.2%)	36(54.5%)	14(21.2%)	2(3%)	0	0	66

Note: number (percentage).

**Table 3:** The width sample distribution of unshod feet and shod feet.

Unit: (mm)		<90	90–100	100–110	110–120	120–130	130<	Sum.
Unshod feet	Male	0	12(13.3%)	48(53.3%)	6(6.7%)	6(6.7%)	18(20%)	90
	Female	0	0	18(23.1%)	30(38.5%)	12(15.3%)	18(23.1%)	78
Shod feet	Male	0	20(15.4%)	50(38.5%)	38(29.2%)	16(12.3%)	6(4.6%)	130
	Female	4(6.1%)	26(39.4%)	18(27.2%)	12(18.2%)	4(6.1%)	2(3%)	66

Note: number (percentage).

For foot length, the unshod feet are in a relatively focused range, with 60% male in the 250–260mm group and 46.2% and 30.8% female in the 230–240mm and 240–250mm groups, respectively. In contrast, the shod male feet are in a distributed range, with similar percentages in 240–250mm (21.5%), 250–260mm (30.8%), 260–270mm (21.5%) and above 270mm (20%) groups. The shod female feet are more fixed in a smaller range than unshod female feet, with 54.5% in the 230–240mm group.

Concerning foot width, the unshod feet show a concentrated range, with 53.3% male unshod feet in the 100–110mm group and 23.1% and 38.5% female unshod feet in 100–110mm and 110–120mm groups, separately. However, shod feet show a dispersed range, with 38.5% and 29.2% male shod feet in 100–110mm and 110–120mm groups and 39.4% and 27.2% female shod feet in 90–100mm and 100–110mm groups.

### One-way ANOVA of length and width of unshod and shod feet

The one-way ANOVA of foot length and width of unshod and shod feet (Table 4) shows that there is a statistically significant difference in the length and width between females with unshod and shod feet with p = 0.001(<0.01). However, the difference in length and width between males with unshod and shod feet was not significant.

**Table 4:** The one-way ANOVA of length and width of unshod and shod feet (mm).

		Unshod feet	Shod feet	F	p
Length	Male	256.5(12.2)	258.1(12.8)	0.844	0.359
	Female	240.2(9.3)	235.4(7.1)	12.003	0.001*
Width	Male	113.4(12.5)	110.4(10.5)	3.751	0.054
	Female	119.2(11.9)	105.2(13.1)	45.123	**0.001#**

Note: Mean(Standard Deviation);

* indicates significance between length of female unshod feet and shod feet;

# indicates significance between width of female unshod feet and shod feet.

### The LSD-ANOVA of HA (HA’) and D (D’)

The least significant difference ANOVA (LSD-ANOVA) analysis of hallux angle and minimal distance (Table 5) shows there is a statistically significant difference between hallux angle HA and HA’ for both male and female runners (p = 0.001); and there is also a statistically significant difference between D and D’ for both male and female runners (p = 0.001). The hallux angle (HA & HA’) of male and female habitually shod and unshod feet was 8.88° (5.17°) and 3.86° (3.49°), with p = 0.001 (<0.01); F = 64.514; and 13.21° (4.89°) and 2.91° (3.45°), with p = 0.001 (<0.01); F = 218.351, respectively. The minimal distance (D & D’) between the hallux and interphalangeal joint of the second toe of male and female habitually shod feet and unshod feet was 6.28mm (6.64mm) and 23.73mm (13.19mm), with p = 0.001 (<0.01); F = 166.995; and 5.39mm (3.91mm) and 19.38mm (10.25mm), with p = 0.01; F = 109.312, respectively. Combining the hallux angle (HA & HA’) with the minimal distance (D & D’), the hallux angle of habitually shod male and female feet are larger than the HA’ of habitually unshod male and female feet. In contrast, the minimal distance (D) of habitually shod male and female feet are smaller than the distance D’ of habitually unshod male and female feet.

**Table 5:** The LSD ANOVA of hallux angle (deg) and distance between unshod and shod feet (mm).

		Male	Female
Hallux Angle	HA	8.88(5.17)	13.21(4.89)
	HA’	3.86(3.49)	2.91(3.45)
	F	64.514	218.351
	p	0.001*	0.001*
Distance	D	6.28(6.64)	5.39(3.91)
	D’	23.73(13.19)	19.38(10.25)
	F	166.995	109.312
	p	0.001*	0.001*

Note: HA-the hallux angle value of shod feet, HA’-the hallux angle value of unshod feet; D-the minimal distance between hallux and second toe of shod feet; D’-the minimal distance between hallux and second toe of unshod feet,

* indicates significance between unshod and shod feet.

**Fig 3:**
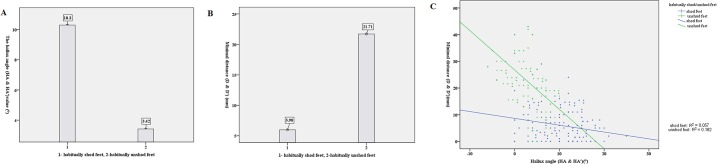
A-The mean value of Hallux Angle (HA = 10.3±5.4 & HA’ = 3.42±3.5) (Fig 3-A), B-minimal Distance (D = 5.98±5.8 & D’ = 21.71±12.1) (Fig 3-B) and C-the correlation between the hallux angle value and the minimal distance with habitually shod feet (R2 = 0.057) and unshod feet (R2 = 0.182) (Fig 3-C).

To illustrate the difference between habitually shod and unshod feet, the hallux angle and minimal distance were analyzed together for both females and males. The mean (SD) value of the hallux angle was HA = 10.3±5.4 and HA’ = 3.42±3.5 (Fig 3A), and the mean (SD) value of the minimal distance was D = 5.98±5.8 and D’ = 21.71±12.1 (Fig 3B). There was a trend observed with the larger the hallux angle the smaller the minimal distance (Fig 3C). However, when quantifying the correlation between hallux angle and minimal distance the fitted values for habitually shod feet (green line) and habitually unshod feet (blue line) were poorly correlated, with R^2^ = 0.057 for habitually shod feet and R^2^ = 0.182 for habitually unshod feet.

## Discussion

Studies concerning foot morphology have been researched ever since the early 20^th^ century [22]. Reasons for morphological differences were attributed to different ethnicities [25,26,28,30], different genders or ages [55], pathological factors [56] and different forms of sport participation [33,35]. In this study, female and male runners of similar age, height and weight or BMI group from China (habitually shod populations) and India (habitually unshod populations) were recruited to illustrate foot morphological characteristics on account of daily footwear wearing and ethnicity influence.

The length of female unshod feet (mean±SD = 240.2±9.3) was significantly larger than that (235.4±7.1) of female shod feet, with p = 0.001, F = 12.003, which was consistent with the length and width (body height) of habitually barefoot Indians who are larger than habitually shod Indians and westerners [25]. However, this was not observed with the male participants in this study, where the difference of length and width was not significant. The explanation for the difference of foot length and width between female participants in this study may be that shod females are more vulnerable to foot deformations, like hallux valgus, owing to wearing high-heeled shoes or sharp-headed shoes [24,48,57]. Long-term wearing of ill-fitted shoes restricted natural foot growth and movement under weight-bearing-conditions [48]. This was observed in extreme cases like the broken longitudinal arch and deformed toes of bound feet in ancient China [23].

The fact that the wearing of poorly-fitted shoes among male participants was seldom may explain the non-significant difference of length and width between habitually shod and unshod feet compared with females [24,57]. A further reason may be attributed to geographic or ethnic influence [28–30,35,55] including wearing slippers or flip-flops [48], sharp-headed shoes [24] or even barefoot. A limitation of the current work which needs to be considered when interpreting the results is that the low overall body height of participants is likely due to different ethnicities. This limits the generalizability to other populations. Studies following on from the current work should consider factors including height (stature), age and BMI are normalized to generalize results to other populations [48,51,54].

The hallux angle and sub-arch index have been proposed to analyze different foot types in previous studies [31,32]. These indices had clearly differentiated hallux angle among different ethnicities (Caucasian, Maori and Pacific Island athletes) [35] and different sub-arch index values of flat foot or high-arched foot [31]. Another useful quantitative index proposed in this study was the minimal distance between hallux and the interphalangeal joint of the second toe. Habitually unshod runners had significantly smaller hallux angle (HA’) and larger minimal distance (D’) than those (HA and D) of habitually shod runners. One feasible explanation for the hallux angle and minimal distance difference between unshod and shod feet was that long-term ill-fitted or sharp-headed shoe wearing adapted the toes shape to a shoe environment (claw-shaped toes) in contrast to their barefoot separate and prehensile function [29,30,48,58]. Moreover, previous studies had pointed out that the separate hallux might work like fingers with prehensile and ambulatory functions [58]. In combination with the difference of HA (HA’) and D (D’), there exists a trend between the hallux angle and the minimal distance in habitually unshod and habitually shod runners, that is, the bigger the HA the smaller the D and the smaller the HA’ the bigger the D’, but these were poorly correlated (R^2^ = 0.057 and R^2^ = 0.182, respectively). The 1.5-million-year-old Hominin footprint revealed morphological characteristics of abducted hallux with hallux abduction angle relative to the foot long axis, showing a difference between abducted hallux and the adducted hallux of modern shoe-wearing feet [29]. The hallux abduction angle is similar to the hallux angle in this study. Another limitation of the study was that the arch index wasn’t calculated to quantitatively investigate whether the arch type influenced the hallux angle and minimal distance though previous study had reported it affected foot length and width and this study had exclude participants with flat or high arch [25].

An application where morphological characteristics of habitually unshod (Indians) feet and habitually shod (Chinese) feet may be useful is informing footwear design, especially for sport in these two large ethnic populations. From vocational athletes aiming to improve sport performance to recreational runners aiming to maintain physical form, running barefoot is an option, especially for habitually shod runners [11,59] and may provide benefits in effective training [8], performance [2], injury prevention [12], and running-economic [60]. Foot measurements are widely accessible due to increased availability and development of foot sensing technology. The morphological characteristics of foot under different conditions, from non-weight bearing, semi to whole body-weight bearing conditions [52,53], different age, gender or specific foot regions [55,61,62] and different ethnicities [28,30,55] have been previously researched. This study shows that measuring hallux angle and distance between hallux and toes is a suitable index to differentiate shod and unshod feet in both males and females.

## Conclusion

Feet morphological characteristics of habitually unshod (Indian) runners and habitually shod (Chinese) runners were analyzed with quantitative indices of feet length and width, the relation in hallux angle and the minimal distance between hallux and the second toe. Quantitative difference exists in terms of female foot length and width. The hallux angle value was greatly correlated with minimal distance from hallux to second toe. One reason for the difference is ethnicity (Chinese and Indian), after accounting for the influence of height, BMI, age and gender. Another reason is that long-term ill-fitted footwear since youth invisibly deformed foot from natural develop. A principal application of this information is informing the design of footwear in the sports industry while considering people from different ethnicities, so as to reduce injuries and improve sports performance. Future study of locomotion biomechanics shall consider the foot morphological characteristics.

## Supporting Information

S1 Fig The participants’ position while foot scanning test (TIF)

S2 Fig Landmarks for the calculation of Hallux Angle (TIF)

S1 Table The participant-level feet morphology data and other relevant information(gender and habitually shod or unshod feet) (DOC)
