# Supplementary figures and images for: Foot Morphological Difference between Habitually Shod and Unshod Runners
Source: PLoS One. 2015 Jul 6;10(7):e0131385. doi: 10.1371/journal.pone.0131385 (PMC4493034; doi:10.1371/journal.pone.0131385)

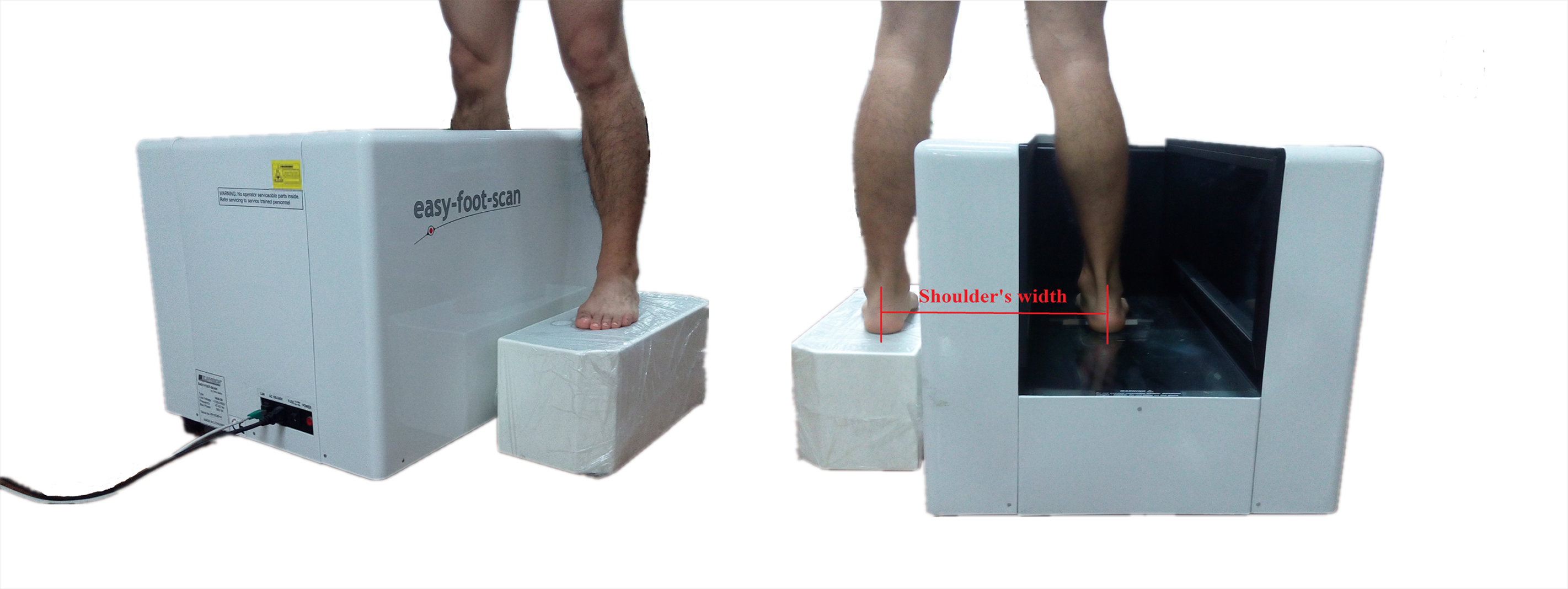

Supplement: S1 Fig — (TIF) [file pone.0131385.s001.tif]

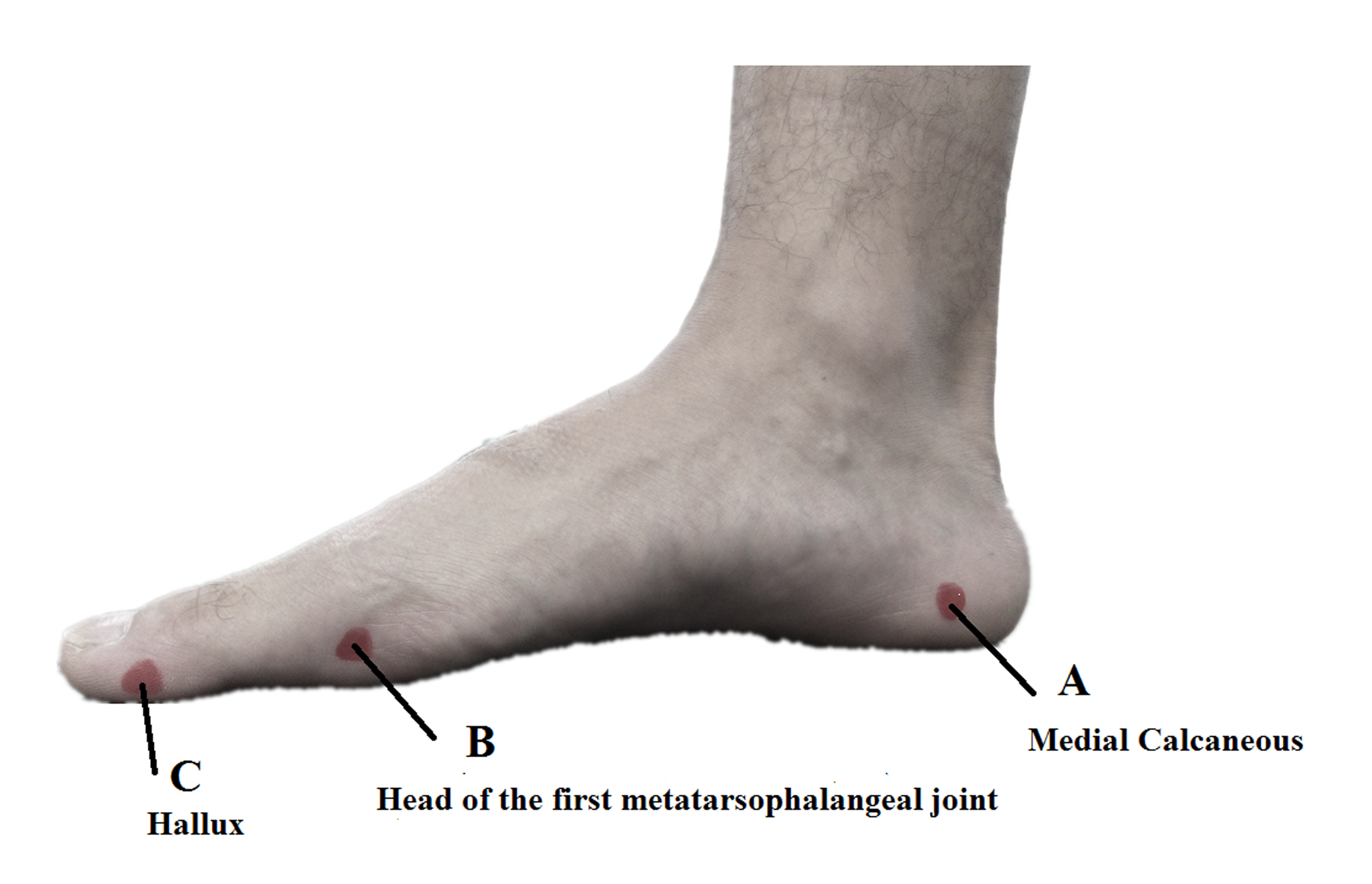

Supplement: S2 Fig — (TIF) [file pone.0131385.s002.tif]
