# Supplementary material for: Foot Morphological Difference between Habitually Shod and Unshod Runners
Source: PLoS One. 2015 Jul 6;10(7):e0131385. doi: 10.1371/journal.pone.0131385 (PMC4493034; doi:10.1371/journal.pone.0131385)
Supplement: S1 Table — (DOC) [file pone.0131385.s003.doc]

**S1 Table. The participant-level feet morphology data and other relevant information (gender and habitually shod or unshod feet)**

| NO. | Hallux angle (°)  (HA & HA’) | Minimal distance (mm)  (D & D’) | Length (mm)  (Length & Length’) | Width (mm)  (Width & Width’) | Gender (1-Male, 2-Female) | 1-Shod feet, 2-Unshod feet |
| --- | --- | --- | --- | --- | --- | --- |
| 1 | 12 | 18 | 277.7 | 97.9 | 1 | 1 |
| 2 | 10 | 1 | 251.9 | 99.2 | 1 | 1 |
| 3 | 1 | 5 | 271.4 | 105.4 | 1 | 1 |
| 4 | 9 | 5 | 274.1 | 109.2 | 1 | 1 |
| 5 | 12 | 11 | 280.5 | 106.8 | 1 | 1 |
| 6 | 14 | 4 | 247.2 | 120.8 | 1 | 1 |
| 7 | 16 | 3 | 249.7 | 105.1 | 1 | 1 |
| 8 | 3 | 2 | 243.6 | 106.7 | 1 | 1 |
| 9 | 15 | 9 | 276.5 | 126.6 | 1 | 1 |
| 10 | 16 | 4 | 259.1 | 101.7 | 1 | 1 |
| 11 | 5 | 12 | 244.9 | 124.2 | 1 | 1 |
| 12 | 21 | 2 | 270.3 | 112.2 | 1 | 1 |
| 13 | 12 | 24 | 295.5 | 134.2 | 1 | 1 |
| 14 | 17 | 0 | 254.4 | 102.3 | 1 | 1 |
| 15 | 16 | 2 | 256.7 | 128.1 | 1 | 1 |
| 16 | 16 | 0 | 285.4 | 104.8 | 1 | 1 |
| 17 | 12 | 2 | 237.6 | 112.6 | 1 | 1 |
| 18 | 7 | 8 | 267.6 | 111.4 | 1 | 1 |
| 19 | 6 | 29 | 262.8 | 100.4 | 1 | 1 |
| 20 | 0 | 19 | 260.7 | 117.6 | 1 | 1 |
| 21 | 6 | 15 | 239.9 | 107.3 | 1 | 1 |
| 22 | 13 | 0 | 245.3 | 141.6 | 1 | 1 |
| 23 | 0 | 3 | 271.2 | 125.5 | 1 | 1 |
| 24 | 11 | 10 | 258.2 | 107.5 | 1 | 1 |
| 25 | 13 | 0 | 259.1 | 95.9 | 1 | 1 |
| 26 | 6 | 0 | 246.8 | 109.5 | 1 | 1 |
| 27 | 13 | 0 | 254.3 | 113.4 | 1 | 1 |
| 28 | 7 | 6 | 254.2 | 118.3 | 1 | 1 |
| 29 | 8 | 9 | 242.7 | 121.6 | 1 | 1 |
| 30 | 3 | 10 | 261.8 | 127.8 | 1 | 1 |
| 31 | 10 | 0 | 240.1 | 96.1 | 1 | 1 |
| 32 | 12 | 3 | 251.9 | 98.2 | 1 | 1 |
| 33 | 3 | 5 | 284.6 | 116.3 | 1 | 1 |
| 34 | 7 | 7 | 245 | 100.1 | 1 | 1 |
| 35 | 9 | 4 | 250.2 | 97.5 | 1 | 1 |
| 36 | 14 | 0 | 272.4 | 117.1 | 1 | 1 |
| 37 | 2 | 10 | 263.3 | 114.8 | 1 | 1 |
| 38 | 3 | 0 | 243.3 | 106.5 | 1 | 1 |
| 39 | 0 | 0 | 260.2 | 103.9 | 1 | 1 |
| 40 | 12 | 0 | 264.2 | 100.8 | 1 | 1 |
| 41 | 7 | 9 | 257.6 | 109.6 | 1 | 1 |
| 42 | 12 | 11 | 254 | 103.1 | 1 | 1 |
| 43 | 15 | 0 | 236.9 | 110.6 | 1 | 1 |
| 44 | 14 | 0 | 242.7 | 112.2 | 1 | 1 |
| 45 | 8 | 7 | 252.4 | 99.1 | 1 | 1 |
| 46 | 0 | 8 | 245.4 | 101.9 | 1 | 1 |
| 47 | 12 | 0 | 250.1 | 122.3 | 1 | 1 |
| 48 | 10 | 16 | 263.3 | 114.8 | 1 | 1 |
| 49 | 3 | 15 | 264.6 | 111.3 | 1 | 1 |
| 50 | 10 | 0 | 257.3 | 103.1 | 1 | 1 |
| 51 | 6 | 11 | 261.3 | 113.6 | 1 | 1 |
| 52 | 4 | 7 | 261.7 | 106.5 | 1 | 1 |
| 53 | 9 | 17 | 253 | 111.7 | 1 | 1 |
| 54 | 8 | 0 | 257.3 | 100.2 | 1 | 1 |
| 55 | 11 | 7 | 251.9 | 99.2 | 1 | 1 |
| 56 | 2 | 23 | 269.1 | 135.1 | 1 | 1 |
| 57 | 8 | 0 | 261.3 | 119.8 | 1 | 1 |
| 58 | 4 | 4 | 254.9 | 118.7 | 1 | 1 |
| 59 | 9 | 4 | 258.1 | 99.1 | 1 | 1 |
| 60 | 4 | 5 | 278.2 | 117.7 | 1 | 1 |
| 61 | 10 | 9 | 273.3 | 117.8 | 1 | 1 |
| 62 | 5 | 4 | 263.1 | 100.1 | 1 | 1 |
| 63 | 0 | 0 | 237.4 | 95.5 | 1 | 1 |
| 64 | 16 | 3 | 245.1 | 103.2 | 1 | 1 |
| 65 | 18 | 6 | 248.7 | 101.3 | 1 | 1 |
| 66 | 12 | 18 | 277.7 | 97.9 | 1 | 1 |
| 67 | 10 | 1 | 251.9 | 99.2 | 1 | 1 |
| 68 | 1 | 5 | 271.4 | 105.4 | 1 | 1 |
| 69 | 9 | 5 | 274.1 | 109.2 | 1 | 1 |
| 70 | 12 | 11 | 280.5 | 106.8 | 1 | 1 |
| 71 | 14 | 4 | 247.2 | 120.8 | 1 | 1 |
| 72 | 16 | 3 | 249.7 | 105.1 | 1 | 1 |
| 73 | 3 | 2 | 243.6 | 106.7 | 1 | 1 |
| 74 | 15 | 9 | 276.5 | 126.6 | 1 | 1 |
| 75 | 16 | 4 | 259.1 | 101.7 | 1 | 1 |
| 76 | 5 | 12 | 244.9 | 124.2 | 1 | 1 |
| 77 | 21 | 2 | 270.3 | 112.2 | 1 | 1 |
| 78 | 12 | 24 | 295.5 | 134.2 | 1 | 1 |
| 79 | 17 | 0 | 254.4 | 102.3 | 1 | 1 |
| 80 | 16 | 2 | 256.7 | 128.1 | 1 | 1 |
| 81 | 16 | 0 | 285.4 | 104.8 | 1 | 1 |
| 82 | 12 | 2 | 237.6 | 112.6 | 1 | 1 |
| 83 | 7 | 8 | 267.6 | 111.4 | 1 | 1 |
| 84 | 6 | 29 | 262.8 | 100.4 | 1 | 1 |
| 85 | 0 | 19 | 260.7 | 117.6 | 1 | 1 |
| 86 | 6 | 15 | 239.9 | 107.3 | 1 | 1 |
| 87 | 13 | 0 | 245.3 | 141.6 | 1 | 1 |
| 88 | 0 | 3 | 271.2 | 125.5 | 1 | 1 |
| 89 | 11 | 10 | 258.2 | 107.5 | 1 | 1 |
| 90 | 13 | 0 | 259.1 | 95.9 | 1 | 1 |
| 91 | 6 | 0 | 246.8 | 109.5 | 1 | 1 |
| 92 | 13 | 0 | 254.3 | 113.4 | 1 | 1 |
| 93 | 7 | 6 | 254.2 | 118.3 | 1 | 1 |
| 94 | 8 | 9 | 242.7 | 121.6 | 1 | 1 |
| 95 | 3 | 10 | 261.8 | 127.8 | 1 | 1 |
| 96 | 10 | 0 | 240.1 | 96.1 | 1 | 1 |
| 97 | 12 | 3 | 251.9 | 98.2 | 1 | 1 |
| 98 | 3 | 5 | 284.6 | 116.3 | 1 | 1 |
| 99 | 7 | 7 | 245 | 100.1 | 1 | 1 |
| 100 | 9 | 4 | 250.2 | 97.5 | 1 | 1 |
| 101 | 14 | 0 | 272.4 | 117.1 | 1 | 1 |
| 102 | 2 | 10 | 263.3 | 114.8 | 1 | 1 |
| 103 | 3 | 0 | 243.3 | 106.5 | 1 | 1 |
| 104 | 0 | 0 | 260.2 | 103.9 | 1 | 1 |
| 105 | 12 | 0 | 264.2 | 100.8 | 1 | 1 |
| 106 | 7 | 9 | 257.6 | 109.6 | 1 | 1 |
| 107 | 12 | 11 | 254 | 103.1 | 1 | 1 |
| 108 | 15 | 0 | 236.9 | 110.6 | 1 | 1 |
| 109 | 14 | 0 | 242.7 | 112.2 | 1 | 1 |
| 110 | 8 | 7 | 252.4 | 99.1 | 1 | 1 |
| 111 | 0 | 8 | 245.4 | 101.9 | 1 | 1 |
| 112 | 12 | 0 | 250.1 | 122.3 | 1 | 1 |
| 113 | 10 | 16 | 263.3 | 114.8 | 1 | 1 |
| 114 | 3 | 15 | 264.6 | 111.3 | 1 | 1 |
| 115 | 10 | 0 | 257.3 | 103.1 | 1 | 1 |
| 116 | 6 | 11 | 261.3 | 113.6 | 1 | 1 |
| 117 | 4 | 7 | 261.7 | 106.5 | 1 | 1 |
| 118 | 9 | 17 | 253 | 111.7 | 1 | 1 |
| 119 | 8 | 0 | 257.3 | 100.2 | 1 | 1 |
| 120 | 11 | 7 | 251.9 | 99.2 | 1 | 1 |
| 121 | 2 | 23 | 269.1 | 135.1 | 1 | 1 |
| 122 | 8 | 0 | 261.3 | 119.8 | 1 | 1 |
| 123 | 4 | 4 | 254.9 | 118.7 | 1 | 1 |
| 124 | 9 | 4 | 258.1 | 99.1 | 1 | 1 |
| 125 | 4 | 5 | 278.2 | 117.7 | 1 | 1 |
| 126 | 10 | 9 | 273.3 | 117.8 | 1 | 1 |
| 127 | 5 | 4 | 263.1 | 100.1 | 1 | 1 |
| 128 | 0 | 0 | 237.4 | 95.5 | 1 | 1 |
| 129 | 16 | 3 | 245.1 | 103.2 | 1 | 1 |
| 130 | 18 | 6 | 248.7 | 101.3 | 1 | 1 |
| 131 | 18 | 0 | 217.4 | 104.5 | 2 | 1 |
| 132 | 19 | 8 | 240.8 | 103.1 | 2 | 1 |
| 133 | 13 | 4 | 252.2 | 112.3 | 2 | 1 |
| 134 | 10 | 0 | 235.2 | 127.6 | 2 | 1 |
| 135 | 18 | 5 | 240.1 | 106.5 | 2 | 1 |
| 136 | 14 | 6 | 235.3 | 91.9 | 2 | 1 |
| 137 | 15 | 5 | 232.8 | 88.7 | 2 | 1 |
| 138 | 20 | 0 | 232.4 | 106.6 | 2 | 1 |
| 139 | 13 | 5 | 240.2 | 116.5 | 2 | 1 |
| 140 | 14 | 3 | 233.1 | 92.6 | 2 | 1 |
| 141 | 15 | 6 | 237.6 | 92.1 | 2 | 1 |
| 142 | 19 | 5 | 229.7 | 92.1 | 2 | 1 |
| 143 | 8 | 5 | 237.5 | 108.5 | 2 | 1 |
| 144 | 13 | 11 | 232.8 | 107.5 | 2 | 1 |
| 145 | 9 | 15 | 246.7 | 113.2 | 2 | 1 |
| 146 | 17 | 0 | 230.8 | 95.3 | 2 | 1 |
| 147 | 20 | 5 | 233.1 | 93.6 | 2 | 1 |
| 148 | 9 | 7 | 232.2 | 97.6 | 2 | 1 |
| 149 | 10 | 7 | 239.7 | 99.5 | 2 | 1 |
| 150 | 11 | 5 | 238.4 | 89.6 | 2 | 1 |
| 151 | 10 | 4 | 238.4 | 95.2 | 2 | 1 |
| 152 | 10 | 6 | 236.8 | 98.1 | 2 | 1 |
| 153 | 14 | 6 | 218 | 148.1 | 2 | 1 |
| 154 | 15 | 12 | 246 | 94.5 | 2 | 1 |
| 155 | 14 | 11 | 234.2 | 135.4 | 2 | 1 |
| 156 | 15 | 11 | 240.9 | 112.8 | 2 | 1 |
| 157 | 14 | 0 | 241.9 | 98.8 | 2 | 1 |
| 158 | 7 | 0 | 228.4 | 113.8 | 2 | 1 |
| 159 | 5 | 0 | 229.6 | 107.6 | 2 | 1 |
| 160 | 25 | 2 | 239.9 | 99.8 | 2 | 1 |
| 161 | 0 | 8 | 229.6 | 107.6 | 2 | 1 |
| 162 | 10 | 10 | 227.4 | 108 | 2 | 1 |
| 163 | 12 | 6 | 237.9 | 112.2 | 2 | 1 |
| 164 | 18 | 0 | 217.4 | 104.5 | 2 | 1 |
| 165 | 19 | 8 | 240.8 | 103.1 | 2 | 1 |
| 166 | 13 | 4 | 252.2 | 112.3 | 2 | 1 |
| 167 | 10 | 0 | 235.2 | 127.6 | 2 | 1 |
| 168 | 18 | 5 | 240.1 | 106.5 | 2 | 1 |
| 169 | 14 | 6 | 235.3 | 91.9 | 2 | 1 |
| 170 | 15 | 5 | 232.8 | 88.7 | 2 | 1 |
| 171 | 20 | 0 | 232.4 | 106.6 | 2 | 1 |
| 172 | 13 | 5 | 240.2 | 116.5 | 2 | 1 |
| 173 | 14 | 3 | 233.1 | 92.6 | 2 | 1 |
| 174 | 15 | 6 | 237.6 | 92.1 | 2 | 1 |
| 175 | 19 | 5 | 229.7 | 92.1 | 2 | 1 |
| 176 | 8 | 5 | 237.5 | 108.5 | 2 | 1 |
| 177 | 13 | 11 | 232.8 | 107.5 | 2 | 1 |
| 178 | 9 | 15 | 246.7 | 113.2 | 2 | 1 |
| 179 | 17 | 0 | 230.8 | 95.3 | 2 | 1 |
| 180 | 20 | 5 | 233.1 | 93.6 | 2 | 1 |
| 181 | 9 | 7 | 232.2 | 97.6 | 2 | 1 |
| 182 | 10 | 7 | 239.7 | 99.5 | 2 | 1 |
| 183 | 11 | 5 | 238.4 | 89.6 | 2 | 1 |
| 184 | 10 | 4 | 238.4 | 95.2 | 2 | 1 |
| 185 | 10 | 6 | 236.8 | 98.1 | 2 | 1 |
| 186 | 14 | 6 | 218 | 148.1 | 2 | 1 |
| 187 | 15 | 12 | 246 | 94.5 | 2 | 1 |
| 188 | 14 | 11 | 234.2 | 135.4 | 2 | 1 |
| 189 | 15 | 11 | 240.9 | 112.8 | 2 | 1 |
| 190 | 14 | 0 | 241.9 | 98.8 | 2 | 1 |
| 191 | 7 | 0 | 228.4 | 113.8 | 2 | 1 |
| 192 | 5 | 0 | 229.6 | 107.6 | 2 | 1 |
| 193 | 25 | 2 | 239.9 | 99.8 | 2 | 1 |
| 194 | 0 | 8 | 229.6 | 107.6 | 2 | 1 |
| 195 | 10 | 10 | 227.4 | 108 | 2 | 1 |
| 196 | 12 | 6 | 237.9 | 112.2 | 2 | 1 |
| 197 | 8 | 11 | 279.9 | 136.3 | 1 | 2 |
| 198 | 4 | 40 | 258.3 | 109.2 | 1 | 2 |
| 199 | -6 | 28 | 265.8 | 132.8 | 1 | 2 |
| 200 | 7 | 8 | 242.5 | 133.7 | 1 | 2 |
| 201 | 0 | 40 | 259.9 | 109.2 | 1 | 2 |
| 202 | 5 | 27 | 244.3 | 122.2 | 1 | 2 |
| 203 | 6 | 20 | 273.9 | 118.1 | 1 | 2 |
| 204 | 2 | 13 | 227.3 | 106.2 | 1 | 2 |
| 205 | 7 | 5 | 258.8 | 109.2 | 1 | 2 |
| 206 | 4 | 16 | 253.3 | 92.2 | 1 | 2 |
| 207 | 3 | 42 | 259.6 | 109.2 | 1 | 2 |
| 208 | 3 | 35 | 258.8 | 109.2 | 1 | 2 |
| 209 | 7 | 19 | 254.3 | 96.6 | 1 | 2 |
| 210 | 3 | 43 | 258.8 | 109.2 | 1 | 2 |
| 211 | 9 | 9 | 251.9 | 107.7 | 1 | 2 |
| 212 | 7 | 11 | 279.9 | 136.3 | 1 | 2 |
| 213 | 4 | 40 | 258.3 | 109.2 | 1 | 2 |
| 214 | -6 | 28 | 265.8 | 132.8 | 1 | 2 |
| 215 | 7 | 8 | 242.5 | 133.7 | 1 | 2 |
| 216 | 0 | 40 | 259.9 | 109.2 | 1 | 2 |
| 217 | 5 | 27 | 244.3 | 122.2 | 1 | 2 |
| 218 | 6 | 20 | 273.9 | 118.1 | 1 | 2 |
| 219 | 2 | 13 | 227.3 | 106.2 | 1 | 2 |
| 220 | 7 | 5 | 258.8 | 109.2 | 1 | 2 |
| 221 | 4 | 16 | 253.3 | 92.2 | 1 | 2 |
| 222 | 3 | 42 | 259.6 | 109.2 | 1 | 2 |
| 223 | 3 | 35 | 258.8 | 109.2 | 1 | 2 |
| 224 | 7 | 19 | 254.3 | 96.6 | 1 | 2 |
| 225 | 3 | 43 | 258.8 | 109.2 | 1 | 2 |
| 226 | 6 | 9 | 251.9 | 107.7 | 1 | 2 |
| 227 | 8 | 11 | 279.9 | 136.3 | 1 | 2 |
| 228 | 4 | 40 | 258.3 | 109.2 | 1 | 2 |
| 229 | -6 | 28 | 265.8 | 132.8 | 1 | 2 |
| 230 | 7 | 8 | 242.5 | 133.7 | 1 | 2 |
| 231 | 0 | 40 | 259.9 | 109.2 | 1 | 2 |
| 232 | 5 | 27 | 244.3 | 122.2 | 1 | 2 |
| 233 | 7 | 20 | 273.9 | 118.1 | 1 | 2 |
| 234 | 2 | 13 | 227.3 | 106.2 | 1 | 2 |
| 235 | 9 | 5 | 258.8 | 109.2 | 1 | 2 |
| 236 | 4 | 16 | 253.3 | 92.2 | 1 | 2 |
| 237 | 3 | 42 | 259.6 | 109.2 | 1 | 2 |
| 238 | 3 | 35 | 258.8 | 109.2 | 1 | 2 |
| 239 | 7 | 19 | 254.3 | 96.6 | 1 | 2 |
| 240 | 3 | 43 | 258.8 | 109.2 | 1 | 2 |
| 241 | 6 | 9 | 251.9 | 107.7 | 1 | 2 |
| 242 | 8 | 11 | 279.9 | 136.3 | 1 | 2 |
| 243 | 4 | 40 | 258.3 | 109.2 | 1 | 2 |
| 244 | -6 | 28 | 265.8 | 132.8 | 1 | 2 |
| 245 | 7 | 8 | 242.5 | 133.7 | 1 | 2 |
| 246 | 0 | 40 | 259.9 | 109.2 | 1 | 2 |
| 247 | 5 | 27 | 244.3 | 122.2 | 1 | 2 |
| 248 | 3 | 20 | 273.9 | 118.1 | 1 | 2 |
| 249 | 2 | 13 | 227.3 | 106.2 | 1 | 2 |
| 250 | 9 | 5 | 258.8 | 109.2 | 1 | 2 |
| 251 | 4 | 16 | 253.3 | 92.2 | 1 | 2 |
| 252 | 3 | 42 | 259.6 | 109.2 | 1 | 2 |
| 253 | 3 | 35 | 258.8 | 109.2 | 1 | 2 |
| 254 | 7 | 19 | 254.3 | 96.6 | 1 | 2 |
| 255 | 3 | 43 | 258.8 | 109.2 | 1 | 2 |
| 256 | 6 | 9 | 251.9 | 107.7 | 1 | 2 |
| 257 | 5 | 11 | 279.9 | 136.3 | 1 | 2 |
| 258 | 4 | 40 | 258.3 | 109.2 | 1 | 2 |
| 259 | -6 | 28 | 265.8 | 132.8 | 1 | 2 |
| 260 | 7 | 8 | 242.5 | 133.7 | 1 | 2 |
| 261 | 0 | 40 | 259.9 | 109.2 | 1 | 2 |
| 262 | 5 | 27 | 244.3 | 122.2 | 1 | 2 |
| 263 | 3 | 20 | 273.9 | 118.1 | 1 | 2 |
| 264 | 2 | 13 | 227.3 | 106.2 | 1 | 2 |
| 265 | 9 | 5 | 258.8 | 109.2 | 1 | 2 |
| 266 | 4 | 16 | 253.3 | 92.2 | 1 | 2 |
| 267 | 3 | 42 | 259.6 | 109.2 | 1 | 2 |
| 268 | 3 | 35 | 258.8 | 109.2 | 1 | 2 |
| 269 | 7 | 19 | 254.3 | 96.6 | 1 | 2 |
| 270 | 3 | 43 | 258.8 | 109.2 | 1 | 2 |
| 271 | 5 | 9 | 251.9 | 107.7 | 1 | 2 |
| 272 | 8 | 11 | 279.9 | 136.3 | 1 | 2 |
| 273 | 4 | 40 | 258.3 | 109.2 | 1 | 2 |
| 274 | -6 | 28 | 265.8 | 132.8 | 1 | 2 |
| 275 | 7 | 8 | 242.5 | 133.7 | 1 | 2 |
| 276 | 0 | 40 | 259.9 | 109.2 | 1 | 2 |
| 277 | 5 | 27 | 244.3 | 122.2 | 1 | 2 |
| 278 | 3 | 20 | 273.9 | 118.1 | 1 | 2 |
| 279 | 2 | 13 | 227.3 | 106.2 | 1 | 2 |
| 280 | 3 | 5 | 258.8 | 109.2 | 1 | 2 |
| 281 | 4 | 16 | 253.3 | 92.2 | 1 | 2 |
| 282 | 3 | 42 | 259.6 | 109.2 | 1 | 2 |
| 283 | 3 | 35 | 258.8 | 109.2 | 1 | 2 |
| 284 | 7 | 19 | 254.3 | 96.6 | 1 | 2 |
| 285 | 3 | 43 | 258.8 | 109.2 | 1 | 2 |
| 286 | 7 | 9 | 251.9 | 107.7 | 1 | 2 |
| 287 | -4 | 23 | 242.6 | 116.6 | 2 | 2 |
| 288 | 7 | 23 | 247.7 | 135.4 | 2 | 2 |
| 289 | 3 | 21 | 232.3 | 124.9 | 2 | 2 |
| 290 | 4 | 25 | 251.2 | 1124 | 2 | 2 |
| 291 | 5 | 20 | 247.9 | 117.1 | 2 | 2 |
| 292 | 1 | 34 | 235.8 | 138.3 | 2 | 2 |
| 293 | 8 | 14 | 243.9 | 136.6 | 2 | 2 |
| 294 | 0 | 33 | 239.4 | 102.1 | 2 | 2 |
| 295 | 7 | 17 | 233.3 | 128 | 2 | 2 |
| 296 | 2 | 30 | 258.8 | 109.2 | 2 | 2 |
| 297 | 4 | 0 | 235.8 | 112.3 | 2 | 2 |
| 298 | 3 | 8 | 223.9 | 114.7 | 2 | 2 |
| 299 | 4 | 4 | 230.3 | 102.4 | 2 | 2 |
| 300 | -4 | 23 | 242.6 | 116.6 | 2 | 2 |
| 301 | 7 | 23 | 247.7 | 135.4 | 2 | 2 |
| 302 | 3 | 21 | 232.3 | 124.9 | 2 | 2 |
| 303 | 4 | 25 | 251.2 | 1124 | 2 | 2 |
| 304 | 5 | 20 | 247.9 | 117.1 | 2 | 2 |
| 305 | 2 | 34 | 235.8 | 138.3 | 2 | 2 |
| 306 | 8 | 14 | 243.9 | 136.6 | 2 | 2 |
| 307 | 0 | 33 | 239.4 | 102.1 | 2 | 2 |
| 308 | 10 | 17 | 233.3 | 128 | 2 | 2 |
| 309 | 1 | 30 | 258.8 | 109.2 | 2 | 2 |
| 310 | 4 | 0 | 235.8 | 112.3 | 2 | 2 |
| 311 | 3 | 8 | 223.9 | 114.7 | 2 | 2 |
| 312 | 4 | 4 | 230.3 | 102.4 | 2 | 2 |
| 313 | -4 | 23 | 242.6 | 116.6 | 2 | 2 |
| 314 | 7 | 23 | 247.7 | 135.4 | 2 | 2 |
| 315 | 3 | 21 | 232.3 | 124.9 | 2 | 2 |
| 316 | 4 | 25 | 251.2 | 1124 | 2 | 2 |
| 317 | 5 | 20 | 247.9 | 117.1 | 2 | 2 |
| 318 | 2 | 34 | 235.8 | 138.3 | 2 | 2 |
| 319 | 8 | 14 | 243.9 | 136.6 | 2 | 2 |
| 320 | 0 | 33 | 239.4 | 102.1 | 2 | 2 |
| 321 | 6 | 17 | 233.3 | 128 | 2 | 2 |
| 322 | 2 | 30 | 258.8 | 109.2 | 2 | 2 |
| 323 | 4 | 0 | 235.8 | 112.3 | 2 | 2 |
| 324 | 3 | 8 | 223.9 | 114.7 | 2 | 2 |
| 325 | 4 | 4 | 230.3 | 102.4 | 2 | 2 |
| 326 | -4 | 23 | 242.6 | 116.6 | 2 | 2 |
| 327 | 7 | 23 | 247.7 | 135.4 | 2 | 2 |
| 328 | 2 | 21 | 232.3 | 124.9 | 2 | 2 |
| 329 | 4 | 25 | 251.2 | 1124 | 2 | 2 |
| 330 | 5 | 20 | 247.9 | 117.1 | 2 | 2 |
| 331 | 1 | 34 | 235.8 | 138.3 | 2 | 2 |
| 332 | 8 | 14 | 243.9 | 136.6 | 2 | 2 |
| 333 | 0 | 33 | 239.4 | 102.1 | 2 | 2 |
| 334 | 2 | 17 | 233.3 | 128 | 2 | 2 |
| 335 | -3 | 30 | 258.8 | 109.2 | 2 | 2 |
| 336 | 4 | 0 | 235.8 | 112.3 | 2 | 2 |
| 337 | 3 | 8 | 223.9 | 114.7 | 2 | 2 |
| 338 | 4 | 4 | 230.3 | 102.4 | 2 | 2 |
| 339 | -4 | 23 | 242.6 | 116.6 | 2 | 2 |
| 340 | 7 | 23 | 247.7 | 135.4 | 2 | 2 |
| 341 | 1 | 21 | 232.3 | 124.9 | 2 | 2 |
| 342 | 4 | 25 | 251.2 | 1124 | 2 | 2 |
| 343 | 5 | 20 | 247.9 | 117.1 | 2 | 2 |
| 344 | -5 | 34 | 235.8 | 138.3 | 2 | 2 |
| 345 | 8 | 14 | 243.9 | 136.6 | 2 | 2 |
| 346 | 0 | 33 | 239.4 | 102.1 | 2 | 2 |
| 347 | 2 | 17 | 233.3 | 128 | 2 | 2 |
| 348 | -4 | 30 | 258.8 | 109.2 | 2 | 2 |
| 349 | 4 | 0 | 235.8 | 112.3 | 2 | 2 |
| 350 | 3 | 8 | 223.9 | 114.7 | 2 | 2 |
| 351 | 4 | 4 | 230.3 | 102.4 | 2 | 2 |
| 352 | -4 | 23 | 242.6 | 116.6 | 2 | 2 |
| 353 | 7 | 23 | 247.7 | 135.4 | 2 | 2 |
| 354 | 2 | 21 | 232.3 | 124.9 | 2 | 2 |
| 355 | 4 | 25 | 251.2 | 1124 | 2 | 2 |
| 356 | 5 | 20 | 247.9 | 117.1 | 2 | 2 |
| 357 | 0 | 34 | 235.8 | 138.3 | 2 | 2 |
| 358 | 8 | 14 | 243.9 | 136.6 | 2 | 2 |
| 359 | 0 | 33 | 239.4 | 102.1 | 2 | 2 |
| 360 | 3 | 17 | 233.3 | 128 | 2 | 2 |
| 361 | -2 | 30 | 258.8 | 109.2 | 2 | 2 |
| 362 | 4 | 0 | 235.8 | 112.3 | 2 | 2 |
| 363 | 3 | 8 | 223.9 | 114.7 | 2 | 2 |
| 364 | 4 | 4 | 230.3 | 102.4 | 2 | 2 |

A total of 364 participants joined in the foot scanning test, among them (names changed into numbers, NO. for the protection of participants’ privacy, with 1 represents shod/Chinese feet and 2 represent unshod/Indian feet in the seventh row), there are 130 male habitually shod runners, 66 female habitually shod runners and 90 male habitually unshod runners, 78 female habitually unshod runners. Runners here were defined as running or conducting other physical activities with shoes at least three times a week for an hour each time. They all had the history of running outdoors or on the treadmill.

Before the scanning, the participants wrote consent about the procedure and content of the test. Among all the items in written consent, one item was the agreement (YES) or disagreement (NO) about the publication of personal information of foot morphological characteristics. For the protection of participants’ privacy, all individual data were listed in numbers (NO.). The hallux angle value of habitually unshod feet with ‘-’(minus) indicates the Line _B’-C’_ exceed Line _A’-B’_.
